# Time-Resolved Analysis of a Highly Sensitive Förster Resonance Energy Transfer Immunoassay Using Terbium Complexes as Donors and Quantum Dots as Acceptors

**DOI:** 10.1155/2007/79169

**Published:** 2007-11-12

**Authors:** Niko Hildebrandt, Loïc J. Charbonnière, Hans-Gerd Löhmannsröben

**Affiliations:** ^1^Physikalische Chemie, Institut für Chemie und Interdisziplinäres Zentrum für Photonik, Universität Potsdam, Karl-Liebknecht-Straße 24-25, Potsdam-Golm 14476, Germany; ^2^Laboratoire de Chimie Moléculaire, Ecole Européenne de Chimie, Polymères, Matériaux (ECPM), UMR 7509 CNRS, 25 rue Becquerel, 67087 Strasbourg Cedex, France

## Abstract

CdSe/ZnS core/shell quantum dots (QDs) are used as efficient Förster Resonance Energy Transfer (FRET) acceptors in a time-resolved immunoassays with Tb complexes as donors providing a long-lived luminescence decay. A detailed decay time analysis of the FRET process is presented. QD FRET sensitization is evidenced by a more than 1000-fold increase of the QD luminescence decay time reaching ca. 0.5 milliseconds, the same value to which the Tb donor decay time is quenched due to FRET to the QD acceptors. The FRET system has an extremely large Förster radius of approx. 100 Å and more than 70% FRET efficiency with a mean donor-acceptor distance of ca. 84 Å, confirming the applied biotin-streptavidin binding system. Time-resolved measurement allows for suppression of short-lived emission due to background fluorescence and directly excited QDs. By this means a detection limit of 18 attomol QDs within the immunoassay is accomplished, an improvement of more than two orders of magnitude compared to commercial systems.

## 1. INTRODUCTION

CdSe/ZnS semiconductor nanocrystals or quantum dots (QDs) possess unrivalled photophysical properties, such as size tunable emission wavelengths, extremely high extinction coefficients over a broad absorption spectrum and enhanced photostability compared to organic fluorophores [1–4]. Moreover, several concepts have been introduced in order to develop water soluble and biocompatible QDs [5–9]. Homogeneous Förster resonance energy transfer (FRET) immunoassays with QDs as energy acceptors are of particular interest because of the extremely high-extinction coefficients of the QDs over a broad absorption spectrum. This special optical property gives rise to large Förster radii leading to efficient FRET over long distances [10–12]. However, the use of QDs as FRET acceptors with organic dye donors is problematic, probably due to the short-lived emission of these donors, hence FRET could not be shown for these donor-acceptor pairs [13]. Only very few publications deal with QDs as acceptors within the biological context, for example, by using bioluminescence energy transfer [14, 15], or with FRET donors of Tb and Eu complexes [16, 17]. In this contribution, we extend these investigations by a thorough analysis of donor and acceptor luminescence decay times, which are important parameters for understanding the dynamic parameters of the FRET process [11, 18]. Within a fluoroimmunoassay of a Tb complex streptavidin conjugate and biotinylated QDs, the sensitized QD acceptor as well as the Tb donor should change their luminescence decay times once they are brought to close proximity by the biotin-streptavidin binding process. For both QD as well as Tb luminescence decay times, we provide further evidence of efficient QD sensitization by FRET from Tb. Previously reported large Förster radii, the high FRET efficiency, and the assumed biotin-streptavidin binding model are confirmed. Moreover, taking advantage of the time-resolved measurement for suppressing the short-lived background emission and QD fluorescence (from directly excited QDs), and optimizing laser excitation (new laser system with low background emission) and solvent conditions (azide-free solvent leading to decreased luminescence quenching of Tb), a very low detection limit is obtained. This means that a sensitivity improvement of more than two orders of magnitude is accomplished, taking the well established and extensively studied Eu-TBP (Eu^3+^-tris(bypyridine) and APC (allophycocyanin) donor-acceptor system [19–21], used within the same streptavidin-biotin assay format, for comparison. The presented results demonstrate the great potential of the Tb to QD FRET system for highly sensitive homogeneous immunoassays for biological as well as clinical and medical applications.

## 2. MATERIALS AND METHODS

### 2.1. FRET donors and acceptors

The FRET donors are conjugates of the tetrameric protein streptavidin (Strep) labeled with Tb complexes (Tb**L**), produced as described in the literature [17, 22]. A labeling ratio of (10 ± 1) Tb**L** per Strep was determined by UV-Vis absorption spectroscopy [22].

The FRET acceptors are commercially available biotinylated CdSe/ZnS core/shell quantum dot nanocrystals emitting at 655 nm (Biot-QD) purchased from Invitrogen Corporation (Carlsbad, Calif, USA). A ratio of ca. 6 biotin molecules per QD is specified by the supplier.

Strep has a very high binding affinity towards biotin with a first dissociation constant of the complex of 10^−13^ M [23]. The biological FRET system is obtained by the strong recognition process between biotin and streptavidin leading to a close proximity of donor and acceptor.

### 2.2. Fluoroimmunoassay

The fluoroimmunoassay (FIA) was performed by adding increasing amounts (0–150 μl) of Biot-QD stock solution (concentration c = $1\cdot 10^{-9}$ M) to a stock of $1\cdot 10^{-9}$ M Tb**L**-Strep (150–0 μl) leading to a total assay volume of 150 μl for each Tb**L**-Strep + Biot-QD mixture. The used solvent was 50 mM borate buffer (pH 8.3) with 2% bovine serum albumin (BSA) and 0.5 M potassium fluoride (KF).

The assay was excited at 315 nm by a Nd:YAG-OPO laser system (Nd:YAG-Laser: Spectra-Physics, Mountain View, Calif, USA; OPO: GWU-Lasertechnik, Erftstadt, Germany) working at 20 Hz repetition rate, with an average pulse energy of ca. 15 μJ, fibre coupled to the fluoroimmunoreader.

The reader system is a commercially available Kryptor immunoreader (Cezanne, Nîmes, France) modified for 315 nm excitation wavelength. Luminescence intensities were collected at (665±5) nm (channel A – QD emission) and at (545±5) nm (channel B – Tb emission). Time-resolved detection is performed by single photon counting with 2 microseconds integration steps over 8 milliseconds using one photon multiplier tube (PMT) for each channel [22].

### 2.3. Time-resolved FRET calculations

Luminescence decay curves for the different mixtures of Tb**L**-Strep + Biot-QD are collected for both QD (channel A) and Tb (channel B) luminescence.

The time-dependent luminescence intensity in channel A (*I*_A_(*t*)) is the sum of a background emission (due to directly excited QDs (*I*(BgQD)), a weak Tb emission (*I*(BgTb)) occuring from the ${}^{5}{\text{D}}_{4}\to {}^{7}{\text{F}}_{2-0}$ transitions) and the QD emission arising from Tb to QD energy transfer given by FRET theory [11]:

(1) $$\begin{array}{ll} I_{\text{A}}(t) & =a\cdot I(\text{BgQD})+b\cdot I(\text{BgTb})\\ & \;+c\cdot \text{exp}\left[-\frac{t}{\tau_{\text{D}}}-\frac{t}{\tau_{\text{D}}}\cdot {\left(\frac{R_{0}}{r}\right)}^{6}\right], \end{array}$$

where $\tau_{\text{D}}$ is the Tb luminescence decay time of pure Tb**L**-Strep (absence of the QD acceptor), *R*_0_ is the Förster radius of the donor-acceptor pair, *c* is the amount of transfered energy or FRET intensity, and *r* is the average donor-acceptor distance. For the decay curves obtained from our experiments, *I*(BgQD) is the time-dependent luminescence intensity of 0.1 nM pure Biot-QD in channel A, *I*(BgTb) is the time-dependent luminescence intensity of 1 nM pure Tb**L**-Strep in channel A, and *a* and *b* are correction factors depending on the actual concentration of Biot-QD and Tb**L**-Strep (including donor emission decrease due to FRET) within the different mixtures.

The two variable parameters *r* and *c* are fitted (using the Microsoft Excel Solver option) so that ((1) fits the respective decay function of each Tb**L**-Strep + Biot-QD mixture. This means that the donor-acceptor distance *r* and the FRET intensity *c* are determined. Assuming that the luminescence decay time of pure QDs is very fast compared to the decay time of the pure Tb donor, the luminescence decay time of QDs upon FRET sensitization $\left(\tau_{\text{A}\text{D}}\right)$ is the same as the Tb decay time in presence of QD $\left(\tau_{\text{D}\text{A}}\right)$ [24)]. $\tau_{\text{A}\text{D}}$ and $\tau_{\text{D}\text{A}}$ can be calculated using:

(2) $$\frac{1}{\tau_{\text{AD}}}=\frac{1}{\tau_{\text{DA}}}=\frac{1}{\tau_{\text{D}}}+\frac{1}{\tau_{\text{D}}}\cdot {\left(\frac{R_{0}}{r}\right)}^{6}\text{.}$$

In order to verify the fitting procedure used for channel A (QD emission channel), a different method was used for channel B, where a simple bi-exponential fit (using the Origin fit procedure) is performed without using any fixed parameters. The luminescence decay in this channel (Tb emission channel) is determined by $\tau_{\text{D}}$ (no FRET) and $\tau_{\text{D}\text{A}}$ (FRET). QD luminescence does not occur in this channel. The time-dependent luminescence intensity in channel B is given by:

(3) $$I_{\text{B}}(t)=I_{0}+x\cdot \exp\left[-\frac{t}{\tau_{\text{D}}}\right]+y\cdot \exp\left[-\frac{t}{\tau_{\text{DA}}}\right]\text{,}$$

where *I*_0_ is an intensity offset and:

(4) $$\begin{array}{cc} \int_{0}^{\infty }x\cdot \exp\left[-\frac{t}{\tau_{\text{D}}}\right]dt & =x\cdot \tau_{\text{D}}\text{,}\\ \int_{0}^{\infty }y\cdot \exp\left[-\frac{t}{\tau_{\text{DA}}}\right]dt & =y\cdot \tau_{\text{DA}} \end{array}$$

represent the dimensionless luminescence intensities of Tb in absence and presence of QD, respectively. In this case, the FRET intensity can be described by $y\cdot \tau_{\text{D}\text{A}}$ (increasing with Biot-QD addition) or by $x\cdot \tau_{\text{D}}$ (decreasing with Biot-QD addition).

### 2.4. Immunoassay detection limit

For calculation of the limit of detection (LOD) for Biot-QD in this type of FIA, the luminescence intensity ratio (*R*_I_) of channel A and channel B integrated from 0.1 to 1.2 milliseconds after the excitation laser pulse (time gating) was used:

(5) $$R_{I}=\frac{\int_{0.1\text{ms}}^{1.2\text{ms}}I_{\text{A}}\left(t\right)dt}{\int_{0.1\text{ms}}^{1.2\text{ms}}I_{\text{B}}\left(t\right)dt}\text{.}$$

*R*_I_ is displayed as a function of Biot-QD concentration for the different mixtures of Tb**L**-Strep + Biot-QD within the FIA. The linear part of the rising *R*_I_ over [Biot-QD] is then used to calculate the LOD by three times the standard deviation of 12 *R*_I_ values at [Biot-QD] = 0 $\left(\sigma_{0}\right)$ devided by the slope of *R*_I_ over [Biot-QD]:

(6) $$\text{L}\text{O}\text{D}=3\cdot \sigma_{0}\cdot \frac{\Delta [\text{Biot-QD}]}{\Delta R_{\text{I}}}\text{.}$$

## 3. RESULTS AND DISCUSSION

### 3.1. Luminescence decay time analysis

**Figure 1:**
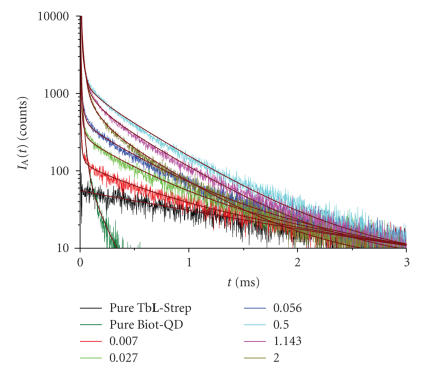
Luminescence decay curves for pure Tb**L**-Strep (c = $1\cdot 10^{-9}$ M) and mixtures of Tb**L**-Strep + Biot-QD with ratios between 0.007 and 2.0 Biot-QD per Tb**L**-Strep measured in channel A ((665±5) nm). A background decay curve of pure Biot-QD (c = $1\cdot 10^{-10}$ M) arising from a strong detector saturation of short-lived directly excited QD fluorescence is also displayed. Fits according to (1) displayed as thick solid lines.

**Figure 2:**
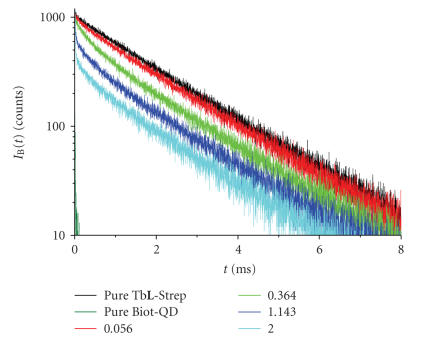
Luminescence and background decay curves (for description, see Figure 1) measured in channel B ((545±5) nm).

Besides the pure Tb**L**-Strep stock solution ($c=1\cdot 10^{-9}$ M) and two pure Biot-QD solutions ($c=1\cdot 10^{-9}$ M and $c=1\cdot 10^{-10}$ M), 13 mixtures of Tb**L**-Strep + Biot-QD with ratios ranging 0.007–2.0 Biot-QD per Tb**L**-Strep were measured. Figures 1 and 2 show some representative luminescence as well as QD background decay curves for the two detection channels.

Regarding the increasing QD emission in channel A (Figure 1), FRET sensitization of QDs by Tb becomes already obvious for low ratios of Biot-QD/Tb**L**-Strep, whereas the QD emission decreases again for ratios higher than 0.5 due to the decreasing Tb**L**-Strep donor concentration with Biot-QD addition (cf. Section 2).

Higher amounts of Biot-QD are necessary for a clearly visible FRET influence in the Tb channel B (Figure 2). There are two reasons for this behavior, the high labeling ratio of 10 Tb**L** per Strep, and the binding of up to six Tb**L**-Strep per Biot-QD, as suggested in previous publications [16, 17, 22]. This means that Biot-QD is saturated with Tb**L**-Strep for low ratios and there is still a majority of free Tb**L**-Strep in solution. Hence, the influence on QD emission is strong whereas it is negligible for Tb. At higher amounts of Biot-QD in the assay, Tb emission is quenched due to FRET and a second decay time becomes obvious in the decay curves.

In order to perform time-resolved FRET calculations using ((1), the Förster radius *R*_0_ (in Å) has to be determined by [11)]:

(7) $$R_{0}^{6}=8.79\cdot 10^{-5}\cdot n_{r}^{-4}\cdot \Phi_{\text{Tb}}\cdot \kappa^{2}\cdot J(\lambda )$$

with:

(8) $$J(\lambda )=\int F_{\text{Tb}}\left(\lambda \right)\cdot {ɛ}_{\text{QD}}\left(\lambda \right)\cdot \lambda^{4}d\lambda \text{,}$$

where *n_r_* is the refractive index of the surrounding medium (1.4 for aqueous media [11]), $\Phi_{\text{T}\text{b}}$ is the Tb centered luminescence quantum yield [25], $\kappa^{2}$ is the dipole orientation factor (2/3 for randomly oriented systems in solution [11, 25]), J(λ) is the overlap integral in M^−1^ cm^−1^ nm^4^, $F_{\text{T}\text{b}}$ is the normalized Tb**L**-Strep luminescence spectrum in nm^−1^ (with $\int F_{\text{T}\text{b}}\left(\lambda \right)d\lambda \;=\;1$), and ${ɛ}_{\text{Q}\text{D}}\left(\lambda \right)$ is the Biot-QD extinction coefficient spectrum in M^−1^ cm^−1^. Taking the luminescence decay time ($\tau^{0}=1.48$ ms) and the quantum yield ($\Phi_{\text{T}\text{b}}^{0}=0.49$) of Tb**L** in pure water [26] and the Tb luminescence decay time of pure Tb**L**-Strep within the assay ($\tau_{\text{D}}=1.83$ milliseconds), a value of $\Phi_{\text{T}\text{b}}=0.61$ can be calculated by [17, 22]:

(9) $$\Phi_{\text{Tb}}=\Phi_{\text{Tb}}^{\text{0}}\cdot \frac{\tau_{\text{D}}}{\tau^{0}}\text{.}$$

**Figure 3:**
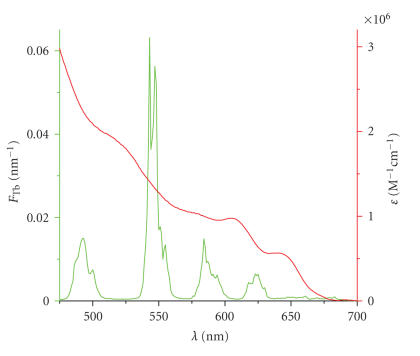
Normalized emission spectrum of Tb**L**-Strep (green) and extinction coefficient spectrum of Biot-QD (red).

Regarding the Tb**L**-Strep luminescence spectrum and the Biot-QD extinction coefficient spectrum in Figure 3, the very good overlap becomes obvious, resulting in an extremely large Förster radius of $R_{0}=\left(100\pm 2\right)$ Å.

Taking this value for fitting *r* and *c* in (1) for each luminescence decay (measured in channel A) of the different Tb**L**-Strep + Biot-QD mixtures leads to a mean donor-acceptor distance of r=(84±1) Å (in good agreement with the proposed Tb**L**-Strep + Biot-QD binding model [16), 17, 22]) and an average FRET sensitized QD luminescence decay time of $\tau_{\text{A}\text{D}}=\left(0.47\pm 0.03\right)$ milliseconds, which is an increase of more than three orders of magnitude compared to the luminescence decay times of pure Biot-QD, which are in the 100-nanosecond range [16, 17].

These values lead to a FRET efficiency of (74±6)% calculated by [11]:

(10) $$\eta_{\text{FRET}}=\frac{R_{0}^{6}}{R_{0}^{6}+r^{6}}=1-\frac{\tau_{\text{AD}}}{\tau_{\text{D}}}\text{.}$$

**Figure 4:**
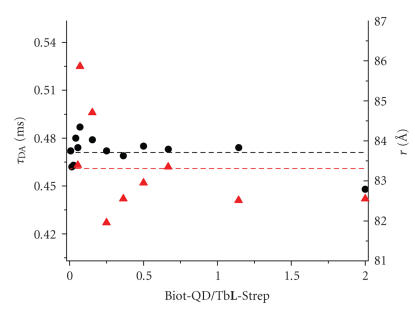
Luminescence decay times of Tb and QDs $\left(\tau_{\text{D}\text{A}}\right)$ due to QD FRET sensitization by Tb and donor-acceptor distances (*r*) calculated from luminescence decay curves of channel A (black dots) and channel B (red triangles), respectively. The dashed lines represent mean values of $\tau_{\text{D}\text{A}}$ and *r*.

The calculations of $\tau_{\text{A}\text{D}}$ and *r* could be confirmed (Figure 4) by fitting the luminescence decay curves of channel B using ((3). Values of $\tau_{\text{D}\text{A}}=\left(0.46\pm 0.07\right)$ milliseconds and r=(83±3) Å are obtained.

As mentioned above, the influence of FRET on the emission signal in channel B starts at higher Biot-QD/Tb**L**-Strep ratios. Hence, only decay curves for ratios above 0.04 are taken into account for the calculations.

Regarding the *r*^−6^ dependence of FRET, it can be assumed that mainly donors which are close to the QD acceptor contribute to the measured FRET signals. As the QD luminescence decay is several orders of magnitude faster than the one of Tb**L**, absorption saturation by direct QD excitation is negligible and a single QD acceptor can interact with several Tb**L** donors. This does not influence the FRET efficiency but lead to a higher FRET signal intensity which has positive impact on assay sensitivity. Nevertheless, due to the distribution of several Tb**L** donors labeled to streptavidin, $\tau_{\text{A}\text{D}}$, *r* and $\eta_{\text{FRET}}$ are average values.

**Figure 5:**
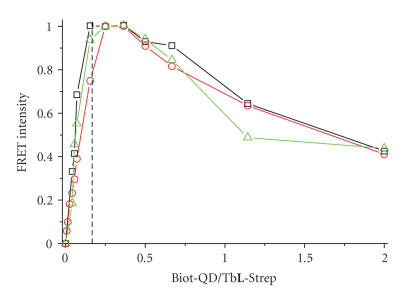
Normalized FRET intensities calculated with (1) and (3) from sensitized QD emission (red circles), short-lived Tb emission due to FRET quenching (black squares), and long-lived Tb emission of unquenched Tb (green triangles). The dotted line indicates a ratio of 6 Tb**L**-Strep per Biot-QD.

Further evidence of FRET from Tb to QD is given by the FRET intensities (*c* from (1) and $x\cdot \tau_{\text{D}}$ as well as $y\cdot \tau_{\text{D}\text{A}}$ from (3)) obtained from fitting the decays of channel A and B. In order to compare the FRET intensities arising from the different signals, they were normalized to unity at a Biot-QD/Tb**L**-Strep ratio of 0.25 and the decreasing (FRET quenched) donor intensity $\left(x\cdot \tau_{\text{D}}\right)$ was subtracted from the concentration-dependent pure Tb**L**-Strep donor signal (Tb signal in absence of QD). Figure 5) shows the very good correlation of the FRET intensities calculated from the three different signals.

A strong increase is first observed, up to the saturation of the six biotins on the QD surface by Tb**L**-Strep and is followed by a decrease due to the reduced donor concentration in the assay volume. This confirms the strong increase of FRET up to a ratio of 1/6 Biot-QD/Tb**L**-Strep and the proposed Tb**L**-Strep + Biot-QD binding model [16, 17, 22]. Moreover, it shows that large QD aggregates bridged by Tb**L**-Strep are not formed at these low concentrations, as it would result in a signal increase up to much higher Biot-QD/Tb**L**-Strep, regarding the number of ten Tb donors per Strep.

For understanding the dynamic parameters of FRET, investigation of luminescence decay times is of great importance. This luminescence decay time analysis over a broad range of Biot-QD/Tb**L**-Strep ratio gives clear evidence of FRET from Tb to QD within the homogeneous streptavidin-biotin immunoassay and confirms the results accomplished with time-gated emission intensity detection in earlier publications [16, 17, 22].

### 3.2. Immunoassay sensitivity

**Figure 6:**
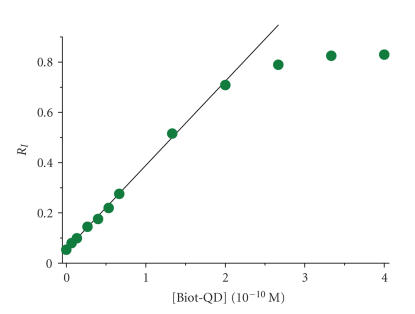
Intensity ratio *R*_I_ (cf. (5)) over Biot-QD concentration for the Tb**L**-Strep + Biot-QD immunoassay after 8 hours incubation. Linear fit for LOD calculation shown as solid line.

In order to evaluate the sensitivity of the assay, *R*_I_ (cf. 5) is displayed over Biot-QD concentration and the LOD is determined from the linear part of the rising *R*_I_ calibration curve obtained from the different Tb**L**-Strep + Biot-QD mixtures (Figure 6).

The *R*_I_ function shows excellent linearity up to a concentration of ca. $2\cdot 10^{-10}$ M Biot-QD. The zero concentration standard deviation has a value of $\sigma_{0}=0.00013$ which leads to an LOD of 0.12 pM Biot-QD (18 attomol of Biot-QD within the 150 μl assay volume) taking the slope of the linear fit in Figure 6).

Comparing this detection limit to earlier results using the same assay with azide containing solutions displays an improvement of one order of magnitude and an LOD decrease of more than two orders of magnitude compared to the homogeneous FRET FIA “gold standard” donor-acceptor pair of Eu-TBP and APC [21] using the same streptavidin-biotin assay [17].

These results underline the very high sensitivity of the Tb to QD FRET system to be used as powerful tool in biomedical analysis, with the possibility of applications in long-distance biological measurements, for example, in high throughput screening for in vitro diagnostics. However, it has to be taken into account that real-life immunoassays can be complicated biological systems and intensive optimization of antibody labeling and measuring conditions is required. First results with good evidence for FRET in a human chorionic gonadotropin (HCG) assay (e.g., applied as pregnancy test) demonstrate the feasibility [22]. In order to achieve the detection limits demonstrated here, these real-life assays are currently under investigation for further optimization.
